# Decreased Striatal RGS2 Expression Is Neuroprotective in Huntington's Disease (HD) and Exemplifies a Compensatory Aspect of HD-Induced Gene Regulation

**DOI:** 10.1371/journal.pone.0022231

**Published:** 2011-07-14

**Authors:** Tamara Seredenina, Ozgun Gokce, Ruth Luthi-Carter

**Affiliations:** Laboratory of Functional Neurogenomics, Ecole Polytechnique Fédérale de Lausanne (EPFL), Lausanne, Switzerland; Emory University, United States of America

## Abstract

**Background:**

The molecular phenotype of Huntington's disease (HD) is known to comprise highly reproducible changes in gene expression involving striatal signaling genes. Here we test whether individual changes in striatal gene expression are capable of mitigating HD-related neurotoxicity.

**Methodology/Principal Findings:**

We used protein-encoding and shRNA-expressing lentiviral vectors to evaluate the effects of RGS2, RASD2, STEP and NNAT downregulation in HD. Of these four genes, only RGS2 and RASD2 modified mutant htt fragment toxicity in cultured rat primary striatal neurons. In both cases, disease modulation was in the opposite of the predicted direction: whereas decreased expression of RGS2 and RASD2 was associated with the HD condition, restoring expression enhanced degeneration of striatal cells. Conversely, silencing of RGS2 or RASD2 enhanced disease-related changes in gene expression and resulted in significant neuroprotection. These results indicate that RGS2 and RASD2 downregulation comprises a compensatory response that allows neurons to better tolerate huntingtin toxicity. Assessment of the possible mechanism of RGS2-mediated neuroprotection showed that RGS2 downregulation enhanced ERK activation. These results establish a novel link between the inhibition of RGS2 and neuroprotective modulation of ERK activity.

**Conclusions:**

Our findings both identify RGS2 downregulation as a novel compensatory response in HD neurons and suggest that RGS2 inhibition might be considered as an innovative target for neuroprotective drug development.

## Introduction

Huntington's disease (HD) is a hereditary neurodegenerative disorder caused by a CAG repeat expansion mutation encoding an abnormally long polyglutamine tract in the huntingtin (htt) protein [1]. Accumulation of mutant htt and its polyglutamine-containing fragments subsequently manifests as nuclear htt enrichment and the formation of intracellular inclusion bodies in neurons of the striatum and cortex, the cells that eventually degenerate in HD [2], [3], [4]. Mutant htt attacks multiple cellular processes crucial for neuronal function [5]; these include transcriptional regulation, energy metabolism, neurotransmission, and axonal transport. One of the earliest detectable effects of mutant htt in the brain is the downregulation of striatal signaling genes [6], [7], [8]. The dysregulated genes notably include multiple components of G-protein-coupled receptor (GPCR) signaling cascades, a finding confirmed in multiple lines of mutant htt-expressing mice [9] and human HD brain [10].

A number of different mechanisms have been proposed to explain HD-induced alterations in gene expression [11]. These draw from evidence that mutant htt interacts with and can disrupt the activities of a large number of transcriptional regulatory proteins. The implicated proteins include DNA-binding transcription factors, transcriptional co-regulators, histone-modifying enzymes, and the basal transcription machinery [11], [12]. The relative contributions of various transcriptional abnormalities as important drivers of the disease process are still under active investigation.

A lesser-explored perspective on altered gene expression in HD is the extent to which differentially expressed genes participate in modulating htt toxicity. While it has been widely assumed that transcriptomic changes reflect direct consequences of mutant htt on gene regulation, this is far from being proven. In fact, some gene expression changes may instead reflect the neuron's activation of autocompensatory mechanisms to counteract mutant htt toxicity [13]. Consistent with this notion, gene expression changes are not uniformly reversed in parallel with the modulation of HD phenotype [14], [15], [16]. In the present study, we sought to identify compensatory HD-related gene expression changes to elucidate novel ways to promote neuronal viability.

Here we identify regulator of G-protein signaling 2 (RGS2) as a novel modifier in primary striatal neuron models of HD. The RGS family of proteins consists of over 30 members characterized by the presence of a conserved ∼120 aa RGS domain necessary and sufficient for binding Gα subunits of heterotrimeric G proteins. RGS2 acts as an attenuator of signal transduction for GPCRs via enhancement of the rate of hydrolysis of GTP to GDP by Gα_q_ [17] and Gα_i_ subunits [18], [19]. RGS2 also interacts directly with adenylyl cyclases 3 and 5 [20], [21] to inhibit the synthesis of cAMP, and modulates MAPK signaling [18], [22].

In addition to identifying RGSs as a possible point of intervention to combat striatal neurodegeneration in HD, the present results suggest that the current view of HD-related effects on neuronal gene expression needs to better account for pro-survival autocompensatory responses.

## Results

The aim of the present study was to identify HD-regulated genes that might function as HD modifiers. We selected genes of interest based on microarray gene expression data from HD brain and HD model systems. A simple meta-analysis of data obtained from human HD brain [10], mouse models of HD [9] and a primary neuron model of HD (PGK-htt171-82Q; [23]) allowed us to choose genes whose expression was decreased in all 3 systems (Table 1). We further narrowed our selection of genes to those that were known or suspected to participate in important striatal neuron signaling processes. The following candidates were subsequently chosen for functional testing.

**Table 1:** Expression of potential gene modifiers in different HD models and in human HD brain.

SystemGene	Human HD		R6/2 6 w		R6/2 12 w		CHL2 22 m		Primary striatal neurons	
	Log2FC	P value	Log2FC	P value	Log2FC	P value	Log2FC	P value	Log2FC	P value
RGS2	-0.54	6.81E-05	-0.37	7.50E-02	-1.16	9.11E-05	-0.73	1.88E-04	-1.19	7.53E-09
RASD2/Rhes	-1.38	9.93E-12	-0.76	3.46E-05	-1.37	1.15E-05	-1.12	2.39E-07	-1.46	6.60E-09
Nnat	-0.35	1.82E-06	-0.69	1.71E-03	-0.56	1.52E-03	-0.40	2.97E-02	-1.24	3.97E-05
Ptpn5/STEP	-1.51	6.30E-10	-0.75	3.37E-05	-1.13	6.73E-06	-0.67	9.48E-06	-0.92	1.94E-07

RNA levels are presented as Log2 fold change (Log2FC) of the HD condition compared to control for each microarray experiment. Human caudate data are from [10], R6/2 and CHL2 data are from [9], primary striatal neuron data are from [23].

a) Striatum-enriched phosphatase (STEP/Ptpn5): STEP is a striatal neuron-enriched tyrosine phosphatase whose activity is responsive to glutamatergic and dopaminergic afferents [24], [25], [26]. Reciprocally, STEP activation has been shown to negatively regulate NMDA receptor stimulation via dephosphorylating and promoting the endocytosis of the NMDA receptor subunit NR2B [27].

b) Ras homolog enriched in striatum (Rhes/RASD2): RASD2 is a member of the large ras superfamily of proteins [28], [29]. Interestingly RASD2 knockout mice show a motor coordination deficit [30] and therefore somewhat resemble HD model animals. Moreover, RASD2 plays an important role in regulating dopamine receptor signaling [31], a neurotransmitter system known to be affected in HD [32], [33].

c) RGS2 is known to be an important regulator of Gα_q_ subunit and Gα_i_ -containing heterotrimeric G-proteins, and adenylyl cyclases [20], [34]. G-protein-coupled receptor signaling and cAMP signaling has been shown in previous studies to be significantly abnormal in HD [7], providing a rationale for testing the ability of RGS2 to compensate for such deficiencies.

d) Neuronatin (NNAT) is expressed during brain development and is believed to regulate neuronal differentiation [35]. Previous data in the neuron-like PC12 cell line suggests potential neuroprotective effects of NNAT [36], leading us to hypothesize that it might do the same in HD model systems.

**Figure 1:**
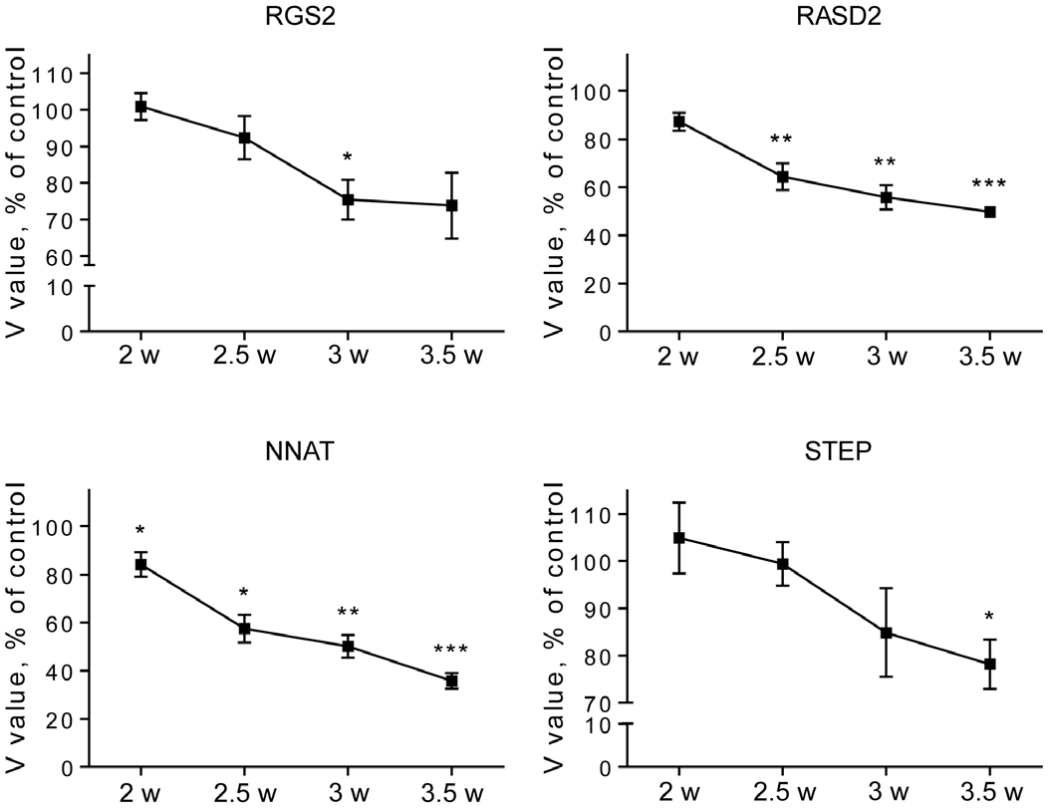
Timecourse of differential expression in the TRE-htt171-82Q primary striatal lentiviral model of HD. RGS2, RASD2, NNAT and STEP RNA levels were measured by qPCR in primary striatal cells expressing mutant htt fragments under the control of a TRE promoter. Target RNA levels (i.e those of RGS2, RASD2, NNAT, STEP) were normalized to the expression level of β-actin and expressed as % control relative to neurons expressing htt171-18Q at the corresponding time point. (mean±SEM); n = 4. * p<0.05; ** p<0.01; *** p<0.001.

We first used qPCR to assess differential expression of the above genes in the TRE-htt171-82Q model of HD [37]. As expected based on HD mouse studies, the qPCR results show progressive decreases in STEP, RASD2, RGS2 and NNAT RNAs in TRE-htt171-82Q compared to TRE-htt171-18Q cells (Fig. 1).

**Figure 2:**
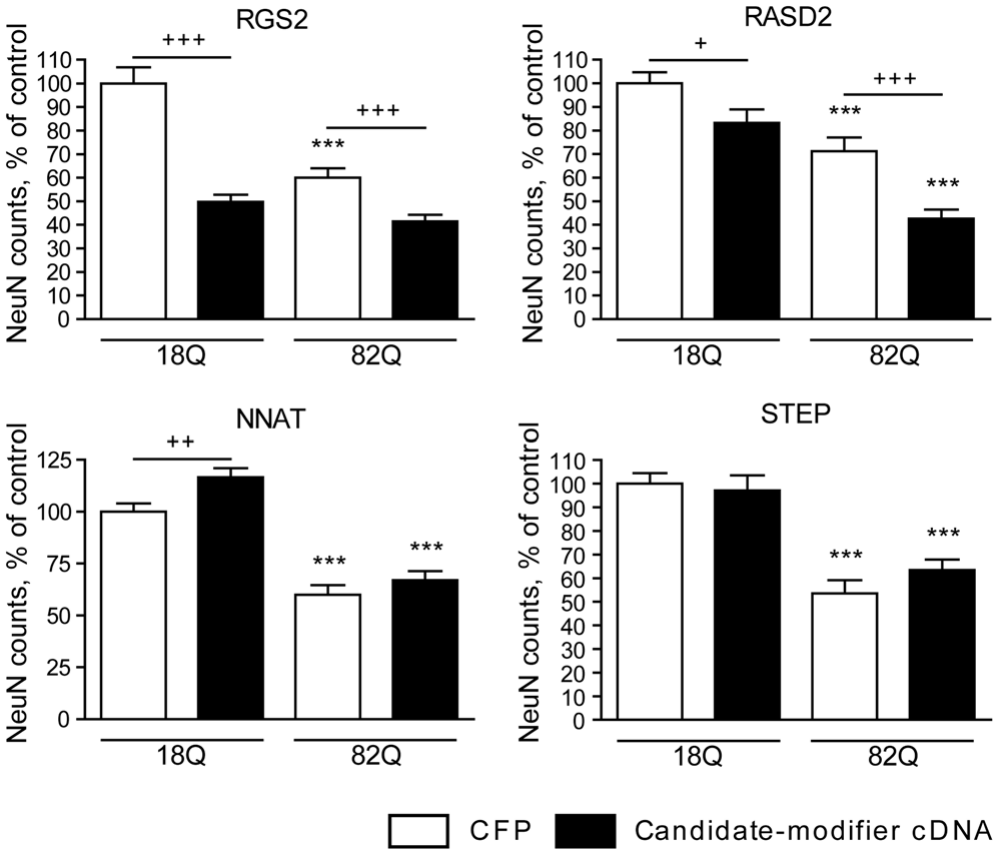
Effects of restoring downregulated RNAs in the TRE-htt171-82Q primary striatal model of HD. Primary striatal neurons were infected on DIV1 with lentiviral vectors encoding WT (18Q) or mutant (82Q) htt171 fragments under the control of a TRE promoter and co-infected on DIV4 with vectors encoding RGS2, RASD2, NNAT, STEP or CFP (control) at a concentration of 25 ng p24 antigen/ml. Neuronal viability was assessed after 3 weeks in culture by quantification of NeuN-positive cells. * indicates significance of mutant htt effects; + indicates significance of effects of the candidate modifier expression. Data are presented as mean±SEM, n = 15, expressed as % control (relative to WT htt171-18Q+CFP). * p<0.05; ** p<0.01; *** p<0.001.

**Figure 3:**
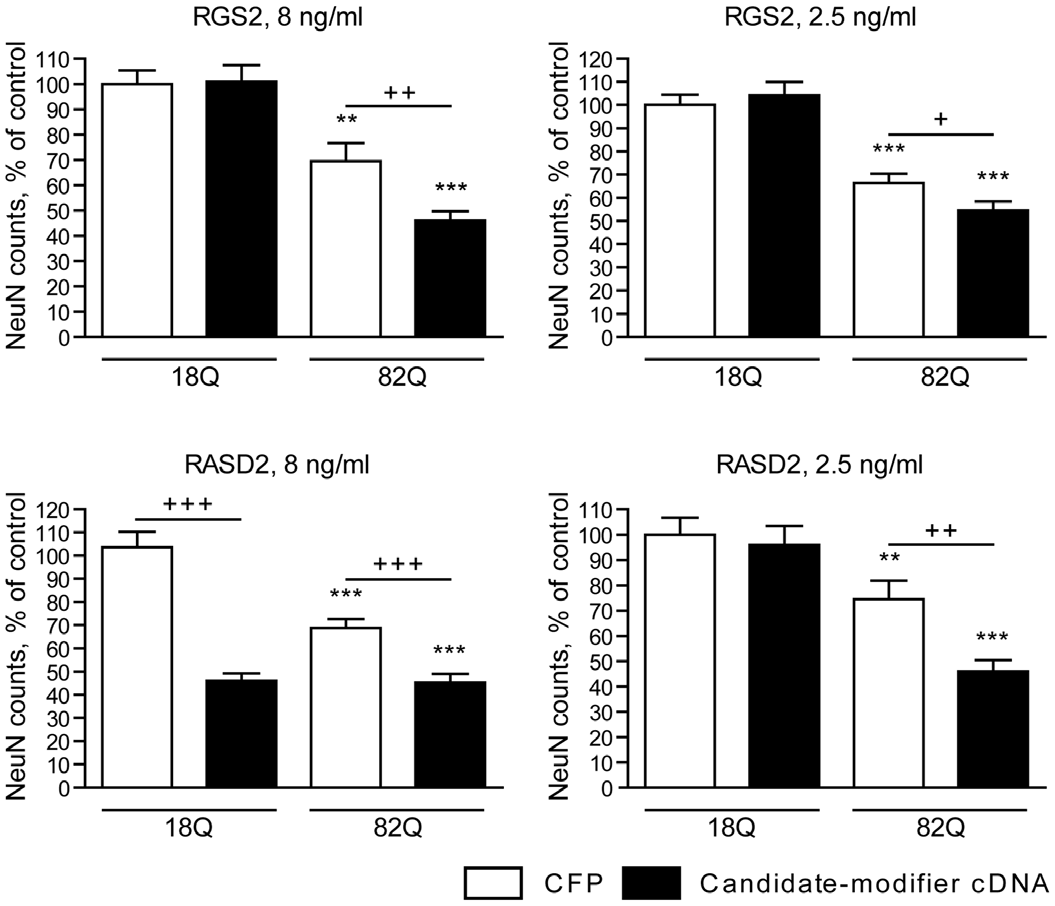
Effects of candidate modifiers at lower expression levels in the TRE-htt171-82Q primary striatal neuron model of HD. Primary striatal neurons were infected on DIV1 with lentiviral constructs encoding WT (18Q) or mutant (82Q) htt171 fragments under the control of a TRE promoter and co-infected on DIV4 with constructs encoding RGS2, RASD2, or CFP (control) at the concentration of 8 or 2.5 ng p24 antigen/ml. Neuronal viability was assessed after 3w in culture by quantification of NeuN-positive cells. * indicates significance of mutant htt effects; + indicates significance of effects of candidate modifier expression. Values are presented as mean±SEM, n = 15, expressed as % control (WT htt171-18Q+CFP). * p<0.05; ** p<0.01; *** p<0.001.

We next tested whether restoring the expression of these genes modified the survival of htt171-82Q-exposed cells. For these experiments, we implemented a primary striatal lentiviral model of HD described previously [37]. As expected, lentivirus-mediated expression of htt171-82Q resulted in a ∼50% decrease in NeuN-positive (neuronal) cells as compared to control cells expressing htt171-18Q (Fig. 2). Co-expression of NNAT or STEP with htt171-82Q did not influence htt toxicity, suggesting that these genes do not have a major disease-modifying effect. In contrast, the expression of RGS2 and RASD2 decreased the viability of neurons expressing either htt171-18Q or htt171-82Q. In order to rule out non-specific viral toxicity as an explanation for the observed effects, we repeated the experiments with independent batches of lentiviral vectors (not shown) and lower vector concentrations (8 ng/ml or 2.5 ng/ml p24 antigen; Fig. 3). These experiments showed that under conditions nontoxic to htt171-18Q cells, both RGS2 and RASD2 still enhanced the toxicity of htt171-82Q. These data indicate that RGS2 and RASD2 may exacerbate HD-related neurotoxicity.

**Figure 4:**
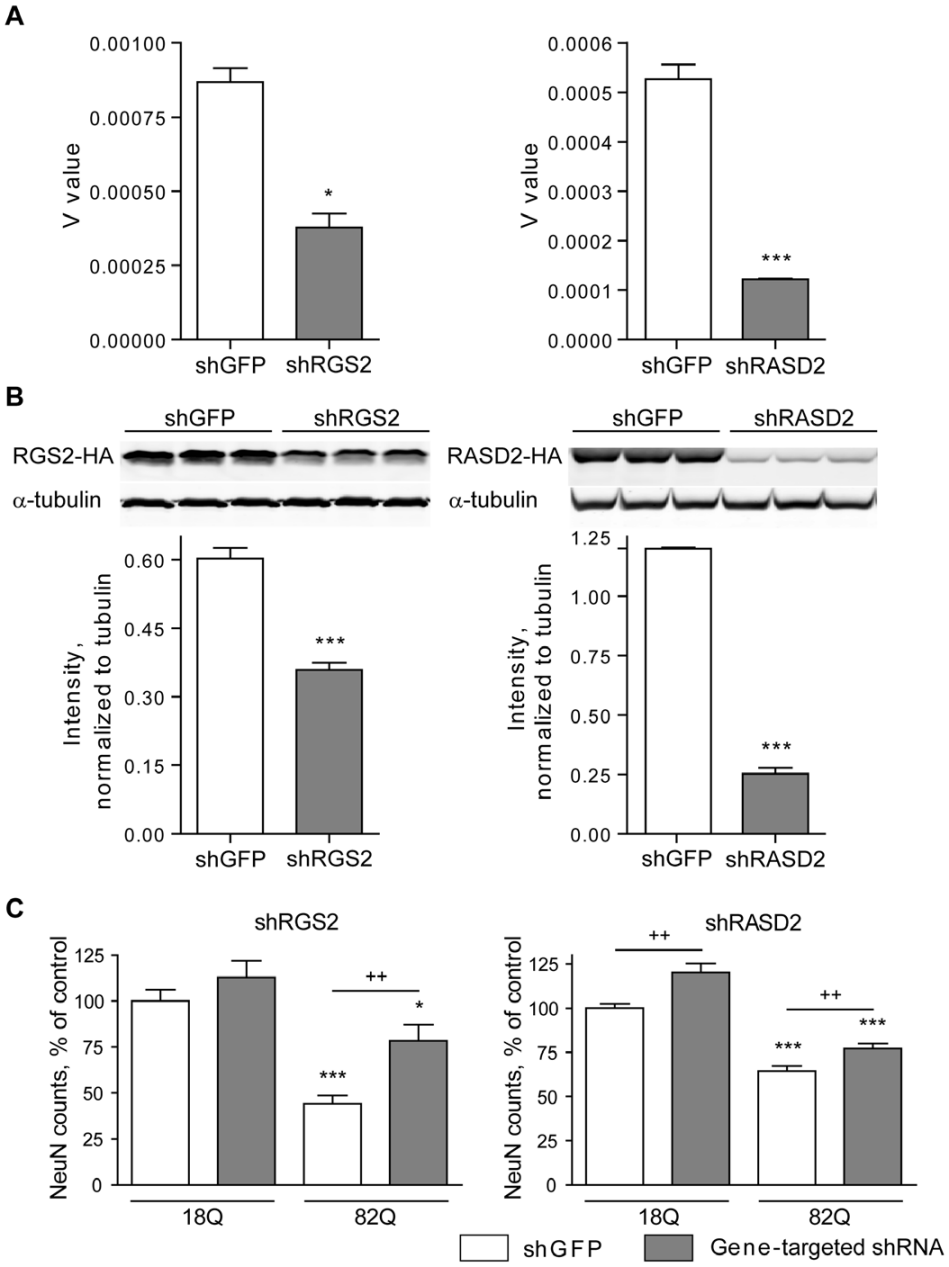
Silencing of RGS2 or RASD2 is protective in the TRE-htt171-82Q primary striatal neuron model of HD. A RGS2-targeting shRNA delivered via a lentiviral vector resulted in a 46.1±7.2% reduction of RGS2 mRNA (in neurons) and 40±5.4% reduction of RGS2 protein expression (in HEK 293T cells), respectively (Panel A). A RASD2-targeting shRNA resulted in a 76.8±0.3% reduction of RASD2 mRNA expression (in neurons) and 79.1±3.6% reduction of RASD2 protein expression (in HEK 293T cells), respectively (Panel B). Immunoblots were performed on HEK 293 cells expressing HA-tagged RGS2 and RGS2 silencing constructs. Lentiviral delivery of an shRNA targeting GFP (shGFP) was used as a control. Values are presented as mean±SEM, n = 3. * p<0.05; ** p<0.01; *** p<0.001. (Panel C). Primary striatal neurons were infected on DIV1 with lentiviral vectors encoding WT (18Q) or mutant (82Q) htt171 fragments under the control of a TRE promoter and co-infected on DIV4 with shRNA vectors targeting RGS2, RASD2, or GFP (control) at a concentration of of 1 ng p 24 antigen/ml. Neuronal viability was assessed by NeuN counting as described in Fig. 2.

The above results suggested to us that decreasing levels of RGS2 and RASD2 might be beneficial to HD neurons. To investigate this hypothesis, we used RNAi to further decrease the expression of RGS2 and RASD2 in parallel with neuronal exposure to htt171-82Q. Lentivirally-delivered shRNAs resulted in effective silencing of both RGS2 and Rhes RNAs and proteins (Fig. 4A,B). Moreover, as predicted, the silencing of both genes protected against htt171-82Q toxicity (Fig. 4C).

**Figure 5:**
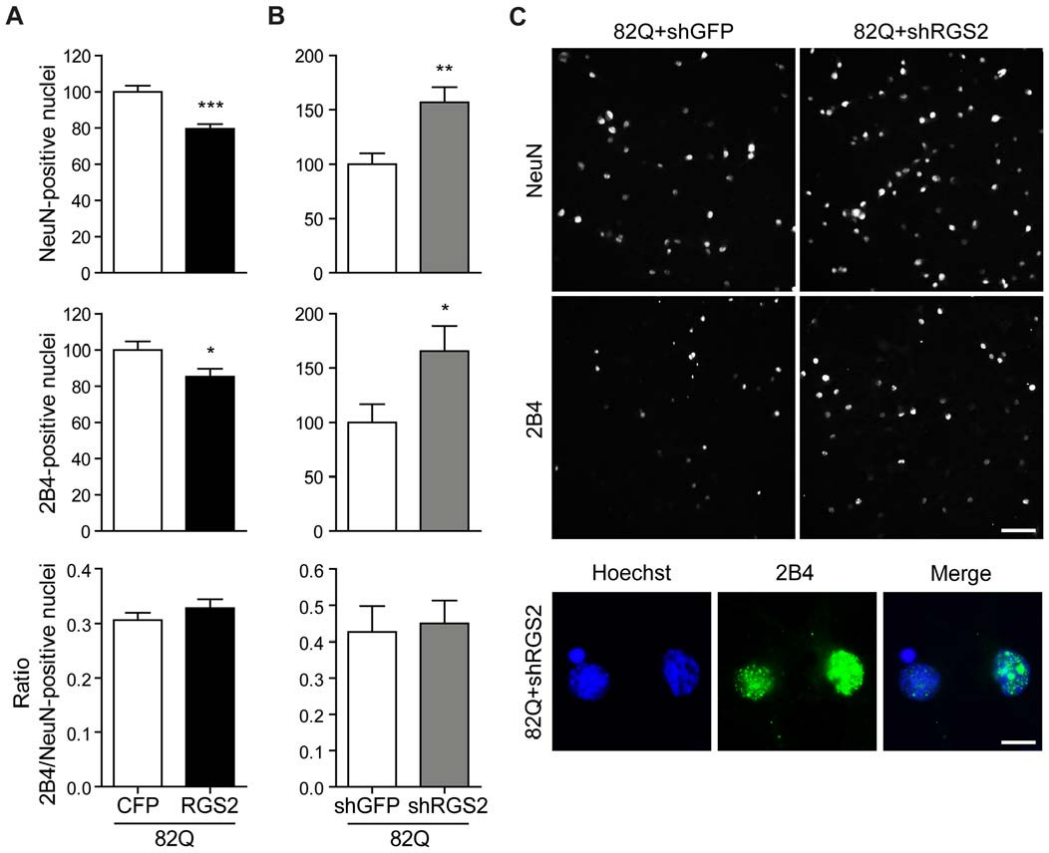
Modulation of RGS2 expression does not influence nuclear accumulation of mutant htt. Primary striatal neurons were infected on DIV1 with lentiviral constructs encoding WT (18Q) or mutant (82Q) htt171 fragments under the control of a TRE promoter and co-infected on DIV4 with an RGS2 expression vector as described in Fig. 2B (A) or RGS2 silencing construct as in Fig. 4B (B). NeuN-positive and 2B4-positive nuclei were quantified as described in the Methods section. (C) Top: representative images of NeuN and 2B4 staining; scale bar 100 µm. Bottom: Representative higher magnification images showing the continued presence of 2B4-positive nuclei after RGS2 silencing; scale bar  = 10 µm.

Whereas a parallel study investigating the role of Rhes in pathogenesis of HD had recently been described [38] (discussed below), the mechanism of modulation of mutant htt toxicity by RGS2 expression was unknown. Therefore, we conducted additional experiments to address this question. We first assessed whether RGS2 expression had an effect on mutant htt levels or the formation of htt inclusions (Fig. 5). Co-expression of RGS2 with mutant htt again resulted in reduced neuronal survival compared to co-expression of mutant htt with CFP as control. Although the total number of mutant htt-positive nuclei was reduced when RGS2 was expressed, this did not represent a change in nuclear accumulation when corrected for neuronal number. Correspondingly, silencing of RGS2 in cells expressing mutant htt increased the number of surviving neurons and the number of nuclei carrying accumulated htt, but not the ratio. These results led us to conclude that modulation of htt toxicity by RGS2 expression levels is not due to a major change in the aggregation or accumulation of mutant htt.

**Figure 6:**
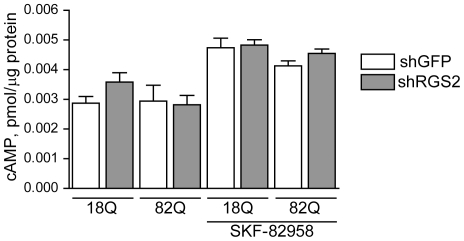
RGS2 silencing does not influence cAMP levels in htt171-82Q-expressing primary striatal neurons. Primary striatal neurons were exposed to htt171-18Q- or htt171-82Q-encoding vectors and RGS2 shRNA (or control GFP shRNA) as described in Fig. 4B. After 2.5 weeks in culture, levels of cAMP were measured in basal conditions and after stimulation with 10 µM SKF-82958 for 15 min. Data are presented as mean±SEM, n = 6.

RGS2 acts as an attenuator of signal transduction for GPCRs via enhancement of the rate of GTP hydrolysis by Gα_i_ [18], [19] which couples negatively to ACs. RGS2 has also been shown to interact directly with AC3 and AC5 to inhibit the synthesis of cAMP [20], [21], [39]. Importantly, cAMP signaling has been a proposed deficit in HD brain [40], [41], and AC5 is the prominent AC in striatum, the most affected HD brain region. We therefore tested the hypothesis that the reduced levels of RGS2 in HD might result in a compensatory restoration in cAMP levels which could explain the RGS2-mediated neuroprotective effect. We first assessed whether modulation of RGS2 expression would influence cAMP levels in striatal neurons in non-disease conditions (Fig. S1). However, neither overexpression nor silencing of RGS2 changed basal cAMP levels (as compared to CFP or shGFP controls, respectively). Stimulation of cAMP biosynthesis with the D1 receptor agonist SKF-82958 resulted in an expected increase in cAMP levels; however, this increase was not influenced significantly by the corresponding expression levels of RGS2 (Fig. S1). We then went on to test whether RGS2 silencing improves cAMP levels in htt171-82Q-exposed neurons. These experiments demonstrated no difference in cAMP level in neurons expressing htt171-82Q compared to htt171-18Q (Fig. 6); moreover, decreasing RGS2 expression was without effect on cAMP levels in either basal or stimulated conditions. We interpret these results to indicate that the protective effect of RGS2 silencing observed in our model is not explained by the modulation of cAMP biosynthesis.

**Figure 7:**
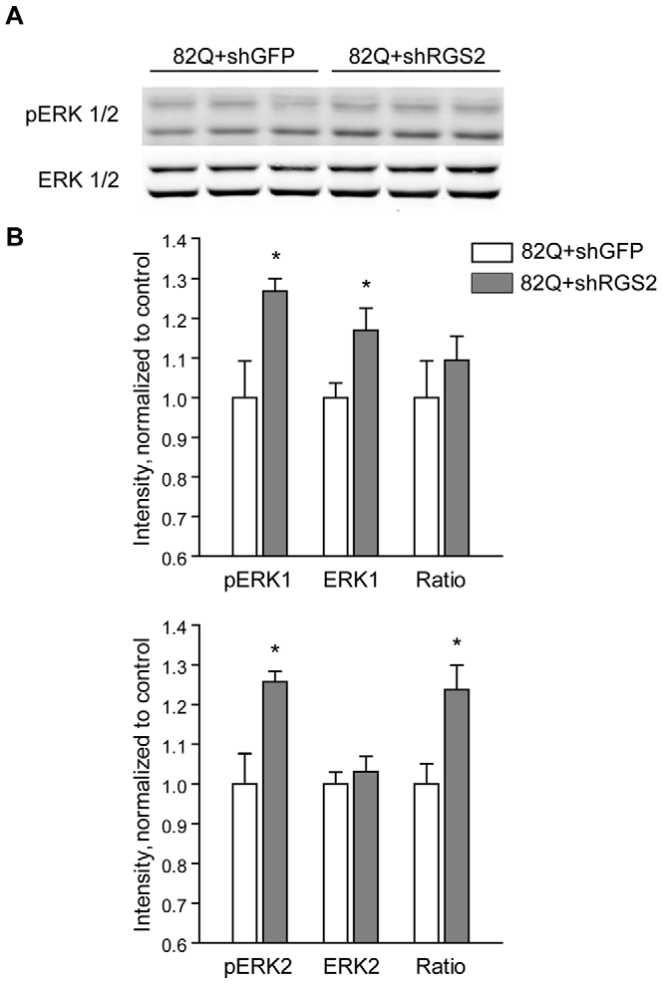
RGS2 silencing increases ERK2 phosphorylation in neurons expressing mutant htt. Primary striatal neurons were infected with htt171-82Q expression and RGS2 silencing vectors as described in Fig. 4B. After 2.5 weeks in culture, levels of ERK1/2 phosphorylation were measured by immunoblot. Data are presented as mean±SEM, n = 6, normalized to htt171-82Q+shGFP control. * p<0.05.

We therefore considered other plausible mechanisms of RGS2-mediated neuroprotection. Previous experiments in COS-7 cells expressing M3 muscarinic receptors showed that RGS2 decreased the phosphorylation and activation of ERK1/2 [18], [22]. Since ERK1/2 activation is a previously implicated mediator of neuroprotection in HD models [42], [43], we tested the hypothesis that inhibiting RGS2 might also be effective by increasing ERK activity. Interestingly, RGS2-silenced cells exposed to mutant htt171-82Q demonstrated the hypothesized increase in ERK1/2 phosphorylation (Fig. 7); a non-statistically significant trend towards increased ERK phosphorylation (after normalization for ERK total protein levels) was also observed in Htt171-18Q cells (Fig. S2). Conversely, Akt phosphorylation was unaffected by RGS2 silencing (Fig. S3). Thus, we suggest that the enhancement of ERK signalling may be the mechanism responsible for the RGS2-silencing-induced neuroprotection.

**Figure 8:**
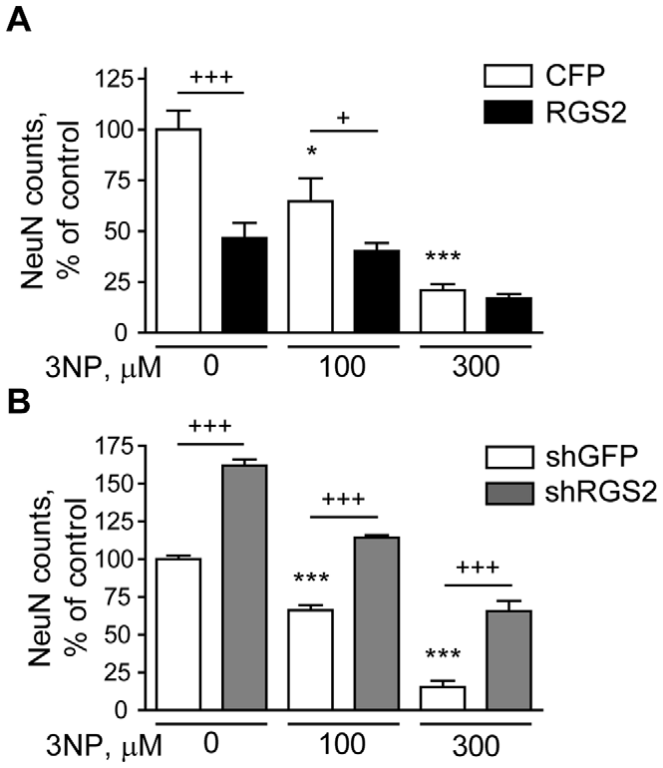
RGS2 levels modulate 3NP toxicity. Primary striatal neurons were infected on DIV 4 with an RGS2 lentiviral expression vector at a concentration of 8 ng/ml p24 (A) or a lentiviral silencing construct targeting RGS2 at concentration of 1 ng/ml p24 (B). Vectors encoding CFP or shGFP were used as controls (in A and B, respectively). After 2 weeks in culture, neurons were treated with 100 or 300 uM 3NP. Neuronal viability was assessed after 48 h by NeuN counting.

Since ERK activation has been shown to be neuroprotective in a number of contexts (for caveats, see Discussion), we also asked whether RGS2 might be able to modulate neurotoxicity due to mitochondrial stress. We therefore assessed its modulation of neuronal viability following exposure of striatal neurons to the mitochondrial toxin 3-nitropropionic acid (3NP). Whereas RGS2 overexpression decreased neuronal viability and enhanced 3NP toxicity, silencing RGS2 protected primary striatal neurons from these effects (Fig. 8). These results suggest that diminishing RGS2 activity may be neuroprotective against a number of toxic stimuli.

Taken together with our previous findings regarding differential gene expression in HD brain, these results indicate that the downregulation of RGS2 and RASD2/Rhes expression comprise autoprotective neuronal responses, rather than etiologic events, in HD. Moreover, the data of the present study provide a novel rationale for exploring RGS modulation of intracellular signalling as a new possible avenue for the development of neuroprotective therapies.

## Discussion

In the present study, we investigated the functional consequences of disease-induced downregulation of the expression of several important striatally expressed genes. The continued evaluation of the downstream effects of HD-induced changes in gene regulation is important to better understand mechanisms of disease pathogenesis and also to identify novel therapeutic targets and pathways that can modulate the course of the disease. The latter is particularly important because no disease-modifying therapy for HD exists to date.

We show in this report that individual gene expression changes have different effects on HD progression, since restoring their expression levels does not uniformly modify mutant htt's effects. Previous studies by our group and others have also suggested that such heterogeneous effects might exist. For example, diminished hippocalcin levels were also shown not to account for sensitization to HD-related toxicities [37], similar to the results observed here for NNAT and STEP.

Importantly, however, we also define another subset of gene expression changes that appears to represent an autocompensatory neuronal response to mutant htt accumulation. One such HD-modifying compensatory response is the downregulation of RASD2/Rhes. An important parallel study implicating Rhes in the pathogenesis of HD was also published recently [38]. Our demonstration that reduction of RASD2/Rhes expression protects striatal neurons from mutant htt is thus concordant with these independently obtained results (in non-neuronal cells). Subramaniam et al. have further provided evidence that Rhes-enhanced sumoylation of mutant htt increases its cytotoxicity. An earlier study suggested that the expression of another striatally enriched gene, CalDAG-GEF1, might account for the brain regional selectivity of mutant htt toxicity [13], by demonstrating that the CalDAG-GEF1 is downregulated in HD human caudate and in R6/2 mice striatum and that silencing of this gene protects neurons from toxicity induced by mutant htt in an organotypic cortico-striatal slice model of HD. Although it is not possible to state for certain that the HD-affected neuron senses and actively compensates for mutant htt’s effects, the end results are clearly beneficial rather than etiologic.

In the present study we identified RGS2 as a novel modifier of HD neurotoxicity. The decreased RGS2 expression observed in HD also seems to be compensatory, since restoration of RGS2 expression enhances mutant htt toxicity, and downregulation of its expression conversely results in neuroprotection. The best-known function of RGS2 is its RGS-domain-mediated attenuation of GPCR-mediated signalling. Our interest in the RGS family of genes was peaked by previous findings that heterotrimeric GPCR signaling is significantly altered in HD [7], [10], [23]. We were somewhat surprised to observe that the modulation of cAMP levels was not a significant contributor to htt toxicity in our model system. However, the HD-induced changes in cAMP levels have been debated, and are likely to comprise a set of potentially complex and dynamic effects [44], [45], [46], [47]. Moreover, some effects attributed to deficient cAMP-related gene regulation invoke downstream steps of transcriptional activation [40], [41] rather than proximal signal transduction events.

We have not ruled out the possible role of RGS2 in regulating other GPCRs important for striatal function. We have determined, however, that negative regulation of RGS2 expression results in an increase in ERK2 phosphorylation that likely explains, at least in part, its neuroprotective effect. Although ERK’s effects on neurodegeneration in other conditions has been debated [48], [49], previous studies have been concordant in showing that ERK activation achieves neuroprotection against HD when ERK is regulated endogenously, regulated by small molecules, or activated via BDNF signaling [42], [43], [50], [51]

Our studies suggest that RGS2 antagonists might be an interesting area for development of anti-neurodegenerative disease therapeutics. This idea is further supported by the fact that RGS2 knock-out mice show increased survival of retinal ganglion cells [52]. However, other studies of RGS2 null-mutant animals suggest proceeding with caution. For example, hypertension and deficiencies in T-cell activation have been reported [53].

Intriguingly, the expression levels of other members of the RGS gene family are also changed in HD models and human HD brain [7], [10], [23]. Since these factors are also potentially important regulators of GPCR signaling, which is impaired in HD, other RGS family members function may also be an interesting subject of further investigation. This is particularly true since this family of proteins is generally considered amenable to small-molecule therapeutics.

## Materials and Methods

### Ethics Statement

All experiments were approved by the local veterinary office and the Commission for Animal Experimentation of the Canton of Vaud Switzerland, authorization number 1667.2 issued by the Service de la consommation et des affaires vétérinaires (SCAV)

### Plasmid expression vectors and lentiviral production

Rat cDNAs encoding RGS2 (NM_053453.2), RASD2 (NM_133568.1), PTPN5 (NM_019253.3) and NNAT transcript variant 2 (NM_181687.1) were amplified from total rat brain cDNA using the polymerase chain reaction (PCR) and cloned into the shuttle plasmid pENTR/D-TOPO (Invitrogen, Carlsbad, CA, USA) using the pENTR Directional TOPO Cloning Kit (Invitrogen). The cDNAs were then transferred from the shuttle vector into a self-inactivating lentiviral plasmid vector with a woodchuck hepatitis virus posttranscriptional regulatory element (SIN-W) under the control of the mouse phosphoglycerate kinase 1 (PGK) promoter using Invitrogen's GatewayTM recombination technology. The same procedure was performed to clone an shRNA-generating construct targeting the rat RASD2 sequence AGAAGAACACGAACGUAAA (with UUCAAGAGA used as the loop sequence). An shRNA-generating construct for rat RGS2 was obtained from Sigma in a pLKO.1-puro vector (targeting sequence GATGAACTGCTGGCCAGTAAA (TRCN0000054762)).

Lentiviral vectors were produced in human embryonic kidney 293T (HEK293T) cells with a four-plasmid system as described previously [37]. The viruses were resuspended in PBS with 1% of BSA and the particle content was assessed by p24 antigen ELISA (RETROtek; Gentaur).

### *In vitro* lentiviral model of Huntington's disease

Primary cultures of striatal neurons were prepared and infected at DIV1 with lentiviral vectors as described in [37]. Htt-expressing lentiviruses encoding the first 171 amino acids of wild-type or mutant human htt, (SIN-W-TRE-htt171-18/82Q, respectively) were applied at a particle concentration of 25 ng p24 antigen/ml culture medium together with a vector encoding the tetracycline-controlled transactivator tTA1 under the control of PGK promoter (at a concentration of 40 ng p24/ml). At DIV4 neurons were infected with lentiviral expression constructs for RGS2, RASD2, NNAT or PTPN5 (at a p24 concentration of 25 ng/ml or 8.3 ng/ml) or with lentiviral silencing constructs for RGS2 and RASD2 (at a p24 concentration of 1 ng/ml). Vectors expressing CFP or an shRNA targeting green fluorescent protein were used as controls for expression and silencing studies, respectively. Three weeks after the infection of htt constructs, cells were fixed and their survival assessed by NeuN counting [37].

### 3-Nitropropionic acid toxicity studies

Primary cultures of striatal neurons were prepared as in [37] and infected at DIV4 with lentiviral constructs for expression or silencing of indicated genes. At DIV17 cultures were treated with 100 or 300 uM 3-nitropropionic acid (3-NP). Forty-eight hours later cells were fixed and their survival assessed by NeuN-positive cell counting.

#### Immunocytochemistry

Cells were fixed in 4% paraformaldehyde for 20 min at RT, washed with PBS and blocked by incubation in PBS containing 10% normal goat serum (NGS, Gibco, Invitrogen, Basel, Switzerland) and 0.1% Triton X-100 (TX, Fluka, Sigma, Buchs, Switzerland). Cells were incubated with primary antibodies for 1h at RT (anti-Neuronal Nuclei (NeuN) (Chemicon MAB377 clone A60; 1∶500) or anti-huntingtin (Chemicon MAB5492 clone 2B4; 1∶500)), washed in PBS and incubated for 1 h at RT with fluorescent secondary antibody (Alexa-Fluor 488, A21121, Invitrogen; 1∶1000). Where indicated, nuclei were stained with Hoechst 33342 dye (Invitrogen, Basel, Switzerland). Images of NeuN-labeled cultures (n = 15 per condition) and of 2B4-labeled cultures (n = 6 per condition) were acquired using fully automated BD Pathway 855 (BD Biosciences, San Diego, USA) microscope under non-saturating exposure conditions, using the same acquisition settings for all samples in a given experiment. Counting of nuclei was performed with ImageJ software (NIH, Bethesda, MD, USA) applying intensity and size thresholds. Neurons with accumulation of htt in the nucleus as determined by 2B4 staining were counted as htt-accumulation positive.

### qPCR

Total RNA was extracted using the RNeasy kit (Qiagen) and 500 ng of total RNA were used for cDNA synthesis employing High Capacity cDNA RT kit (Applied Biosystems). Quantitative real-time PCR was performed on a 7900HT Real-Time PCR System with SDS 2.3 software (Applied Biosystems) using TaqMan Universal PCR Mastermix (Applied Biosystems). Relative expression (V) was calculated by normalization of gene expression to β-actin expression as described in [54]. All qPCR reactions were performed in triplicate. The following TaqMan assays obtained from Applied Biosystems were used: β-actin Rn00667869_m1; RGS2 Rn00584932; RASD2 Rn00592054; NNAT Rn00822063; PTPN5 Rn00570437_m1.

### Statistical analyses

Numerical data represent the mean values ± SEM. The one-way ANOVA was used to compare multiple conditions and the two-tailed Student's t-test was used for two-group comparisons. p < 0.05 was set as the threshold for statistical significance.

### cAMP assay

cAMP measurements were performed using the Direct cAMP ELISA kit (CA-200, Sigma-Aldrich), according to the protocol supplied by the manufacturer. Primary striatal cultures at 2.5 weeks in culture were stimulated with 10 µM Forskolin or 10 µM SKF-82958 for 15 min then harvested by direct lysis in 0.1 M HCl. The supernatant was recovered by centrifugation after which a small aliquot of lysate (10 µl) was used to determine protein concentration using the BCA protein assay (Pierce). The acetylation procedure was employed to increase assay sensitivity. cAMP measurements were normalized to total protein. A minimum of 4 samples per condition were assayed.

### Immunoblot experiments

For analysis of phosphoproteins, primary striatal neurons were harvested in RIPA buffer (Sigma), containing protease inhibitor cocktail (Sigma), phosphatase inhibitor cocktails 1 and 2 (Sigma) and PMSF 1 mM. Proteins were separated on 12% SDS-polyacrylamide gels and transferred to nitrocellulose membrane. Membranes were blocked for one hour in 30% Odyssey® Blocking Buffer in PBS (LI-COR Biosciences) followed by incubation with primary antibodies diluted in Blocking Buffer with 0.1% Tween-20 overnight at 4°C. Membranes were rinsed with 0.1% Tween-20/PBS followed by incubation with secondary antibody diluted in Blocking Solution for one hour at RT. After final rinses, blots were scanned with an Odyssey® Infrared Imager and densitometric measurements were obtained using Odyssey software (LI-COR Biosciences). For the detection of pERK1/2 images were acquired using ImageQuant LAS 4000 (GE Healthcare). Primary and secondary antibodies comprised: mouse anti-phospho-Erk1/2 (Millipore 05-481, 1∶1000), rabbit anti total ERK1/2 rabbit (Millipore 06-182, 1∶1000), rabbit anti-HA tag (Covance MMS-101R, 1∶2000), rabbit anti-a-tubulin (Abcam ab4074, 1∶2000), donkey anti-mouse IRDye 800CW (LI-COR Biosciences, 1∶15000), donkey anti-rabbit IRDye 680 (LI-COR Biosciences, 1∶15000), anti-mouse-HRP (Jackson ImmunoResearch, 1∶10000).

### Human embryonic kidney 293T cell culture and transfection

Human embryonic kidney (HEK) 293T cells (ATCC CRL-11268) were cultured in the DMEM (Invitrogen) with 10% fetal bovine serum and Pen-Strep at 37°C in 5% CO2/air atmosphere. A CaPO_4_-based procedure was used to transfect the cells for RNAi experiments. 0.5 µg of cDNA vectors and 0.02 µg of silencing vectors were used for 2×10^5^ cells. The culture medium was changed 6 hours post-transfection and protein was harvested 48 h later.

## Supporting Information

Figure S1 **cAMP levels are not changed by modulation of RGS2 expression in primary striatal neurons.** Primary striatal neurons were infected with an RGS2-encoding construct as in Fig. 3 (A) or an RGS2 silencing construct as in Fig. 4A (B). After 2 weeks in culture, levels of cAMP were measured in basal conditions and after stimulation with 10 µM SKF-82958 for 15 min. Values are presented as mean±SEM, n = 3. NS – non significant.
(TIF)

Figure S2 **RGS2 silencing effect on ERK2 phosphorylation in neurons expressing wild type htt.** Primary striatal neurons were infected with htt171-18Q expression and RGS2 silencing vectors as described in Fig. 4B. After 2.5 weeks in culture, levels of ERK2 phosphorylation were measured by immunoblot. Data are presented as mean±SEM, n = 5, normalized to htt171-82Q+shGFP control. ** p<0.01.
(TIF)

Figure S3 **RGS2 silencing effect on Akt phosphorylation in neurons expressing mutant htt.** Primary striatal neurons were infected with htt171-82Q expression and RGS2 silencing vectors as described in Fig. 4B. After 2.5 weeks in culture, levels of Akt phosphorylation were measured by immunoblot. Data are presented as mean±SEM, n = 6, normalized to htt171-82Q+shGFP control.
(TIF)
