# Supplementary figures and images for: Decreased Striatal RGS2 Expression Is Neuroprotective in Huntington's Disease (HD) and Exemplifies a Compensatory Aspect of HD-Induced Gene Regulation
Source: PLoS One. 2011 Jul 14;6(7):e22231. doi: 10.1371/journal.pone.0022231 (PMC3136499; doi:10.1371/journal.pone.0022231)

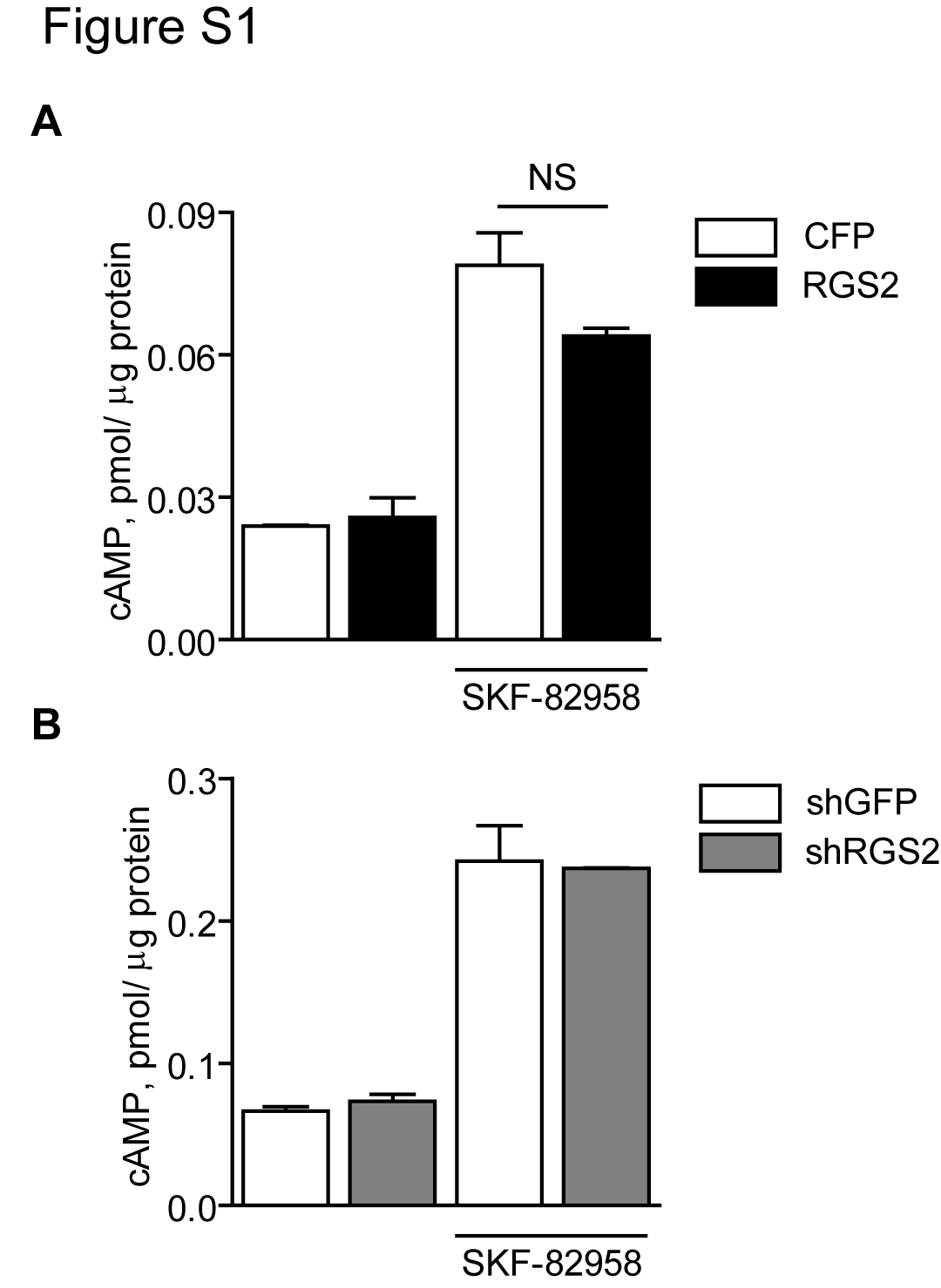

Supplement: Figure S1 — cAMP levels are not changed by modulation of RGS2 expression in primary striatal neurons. Primary striatal neurons were infected with an RGS2-encoding construct as in Fig. 3 (A) or an RGS2 silencing construct as in Fig. 4A (B). After 2 weeks in culture, levels of cAMP were measured in basal conditions and after stimulation with 10 µM SKF-82958 for 15 min. Values are presented as mean±SEM, n = 3. NS – non significant. (TIF) [file pone.0022231.s001.tif]

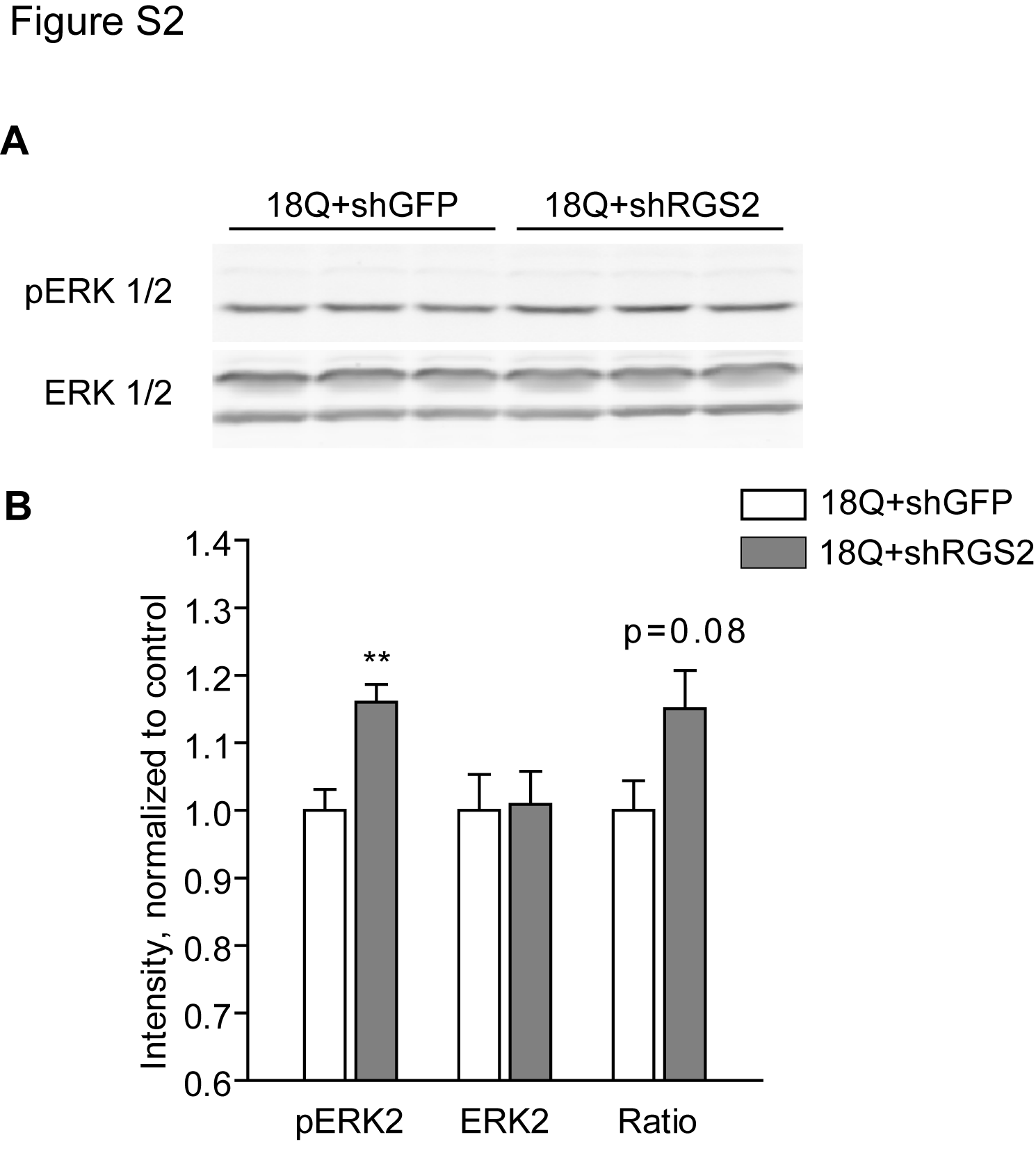

Supplement: Figure S2 — RGS2 silencing effect on ERK2 phosphorylation in neurons expressing wild type htt. Primary striatal neurons were infected with htt171-18Q expression and RGS2 silencing vectors as described in Fig. 4B. After 2.5 weeks in culture, levels of ERK2 phosphorylation were measured by immunoblot. Data are presented as mean±SEM, n = 5, normalized to htt171-82Q+shGFP control. ** p<0.01. (TIF) [file pone.0022231.s002.tif]

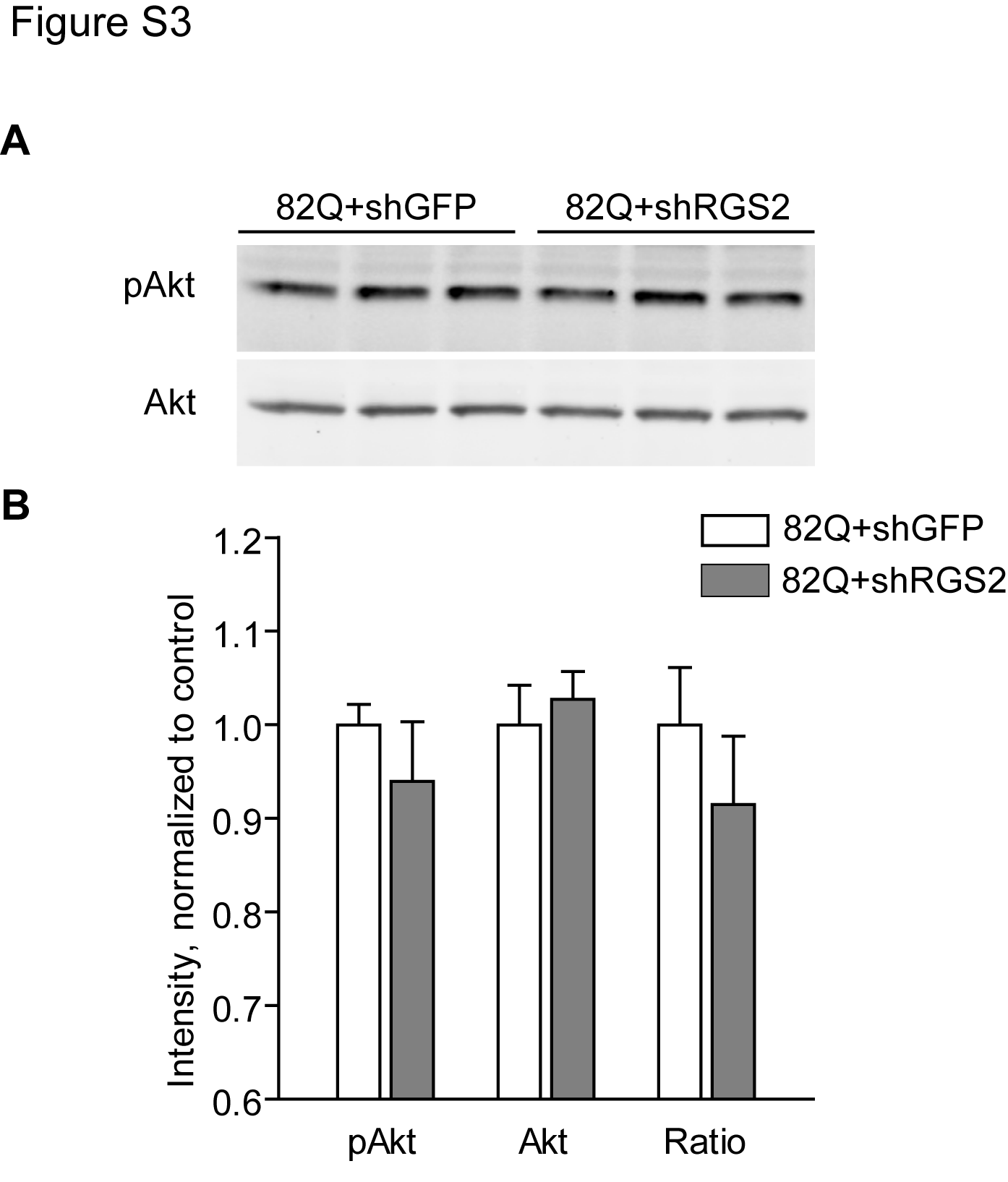

Supplement: Figure S3 — RGS2 silencing effect on Akt phosphorylation in neurons expressing mutant htt. Primary striatal neurons were infected with htt171-82Q expression and RGS2 silencing vectors as described in Fig. 4B. After 2.5 weeks in culture, levels of Akt phosphorylation were measured by immunoblot. Data are presented as mean±SEM, n = 6, normalized to htt171-82Q+shGFP control. (TIF) [file pone.0022231.s003.tif]
